# Health Care Costs Associated With Norovirus at the Veterans Health Administration

**DOI:** 10.1001/jamanetworkopen.2025.36600

**Published:** 2025-10-09

**Authors:** Jordan E. Cates, Richard E. Nelson, Ying Suo, Umesh D. Parashar, Cynthia A. Lucero-Obusan, Mark Holodniy, Sara A. Mirza

**Affiliations:** 1 Division of Viral Diseases, Centers for Disease Control and Prevention, Atlanta, Georgia; 2 Informatics, Decision-Enhancement, and Analytic Sciences Center, Veterans Affairs Salt Lake City, Salt Lake City, Utah; 3 Division of Epidemiology, University of Utah, Salt Lake City; 4 Public Health National Program Office, Department of Veterans Affairs, Washington, DC; 5 Division of Infectious Diseases and Geographic Medicine, Stanford University, Stanford, California

## Abstract

**Importance:**

Norovirus is the leading cause of acute gastroenteritis among all ages in the US. Older adults, including veterans, are a potential target group for future vaccination and therapeutics, and more data are needed on the economic burden of norovirus among this population.

**Objective:**

To quantify the outpatient, emergency department (ED), and inpatient health care costs associated with laboratory-confirmed norovirus episodes among veterans seeking care in the Veterans Health Administration (VHA).

**Design, Setting, and Participants:**

This economic evaluation used VHA electronic health record and health care cost data between January 1, 2010, and December 31, 2024, with costs adjusted to 2024 dollars. Participants were veterans (aged ≥18 years) with norovirus who sought care from VHA clinics and hospitals nationwide. Norovirus episodes were categorized as outpatient, ED, or inpatient.

**Exposure:**

Norovirus episodes laboratory-confirmed by polymerase chain reaction assays.

**Main Outcomes and Measures:**

Outpatient, ED, and inpatient costs for encounters or admissions within 2 days of the norovirus-positive test result. Costs were extrapolated to the expected number of annual norovirus episodes with and without laboratory confirmation in the VHA using published incidence rates.

**Results:**

A total of 7768 norovirus episodes from 7520 patients (median [IQR] age, 62 [45-74] years; 6862 males [88%]; median [IQR] Charlson Comorbidity Index [CCI], 1 [0-4]) were included in the analysis. There were 3520 outpatient, 2018 ED, and 2230 inpatient norovirus episodes, with respective median (IQR) costs of $640 ($207-$1291), $2203 ($1596-$2989), and $14 083 ($8045-$26 672). Median (IQR) inpatient costs were higher among older age groups ($12 777 [$7297-$23 737], $16 075 [$9291-$29 311], and $18 566 [$10 676-$33 582] for those aged 45-64 years, 65-84 years, and ≥85 years, respectively) than younger adults ($7751 [$4768-$14 984] and $9394 [$5474-$15 830] for those aged 18-24 years and 25-44 years, respectively; *P* < .001). Median (IQR) inpatient costs were also higher across CCI categories, with the highest for CCI of 5 or higher ($18 508 [$10 445-$35 900]). The overall extrapolated annual cost to the VHA was $28 438 556.

**Conclusions and Relevance:**

This study found norovirus to be a substantial economic burden in the VHA. Developing targeted interventions, such as vaccines and antivirals, for this population may result in cost savings.

## Key Points

**Question:**

What are the outpatient, emergency department (ED), and inpatient health care costs associated with laboratory-confirmed norovirus episodes among veterans seeking care at the Veterans Health Administration (VHA)?

**Findings:**

In this economic evaluation involving 7768 medically attended norovirus episodes, the median per-episode cost was highest for norovirus episodes that required hospitalization compared with outpatient and ED episodes. The annual extrapolated costs to the VHA were substantial at approximately $28 million.

**Meaning:**

Norovirus has a substantial economic burden in the VHA, and cost savings are possible from developing norovirus vaccines or antivirals for this adult population.

## Teaser

This economic evaluation estimates the costs of norovirus care at the outpatient, emergency department, and inpatient settings of Veterans Health Administration facilities across the US from 2010 through 2024.

## Introduction

Norovirus is the leading cause of acute gastroenteritis among all ages in the US. While norovirus is typically self-limiting within 3 days, older adults and persons with certain medical conditions, such as immunocompromised conditions, are at an increased risk for severe illness.^1,2,3^ Norovirus among adults 65 years or older has been estimated to lead to 424 000 outpatient visits, 60 000 emergency department (ED) visits, 47 000 hospitalizations, and 744 deaths annually in the US.^2,4^

Despite this large disease burden, data quantifying the health care costs among adult populations with laboratory-confirmed norovirus are lacking. Indirect modeling studies estimate that norovirus accounts for up to $1 billion in direct US medical costs among adults 65 years or older, including higher hospitalization costs among adults than children.^4,5,6^ While no norovirus vaccines are currently available, several are in development.^7^ Similarly, there are no approved antivirals for treatment of or prophylaxis against norovirus; however, recent advances in antiviral development are encouraging.^8^ Adult populations are a potential target group for future vaccination efforts and therapeutics, and thus more data are needed on the economic burden among this population.

Economic data on adult medically attended norovirus can contribute to the understanding of health care costs associated with norovirus; inform future cost-effectiveness evaluations; and inform policymakers, funders, and product developers of the potential cost savings from future vaccination and therapeutics in this target population. US veterans represent a group potentially at high risk for norovirus given their older age and high rates of underlying medical conditions.^9,10,11^ In this study, we aimed to quantify the outpatient, ED, and inpatient health care costs associated with laboratory-confirmed norovirus episodes among US veterans seeking care at the Veterans Health Administration (VHA), the largest integrated health care system in the US.^12^

## Methods

### Population and Data Sources

We conducted an economic evaluation using VHA electronic health record (EHR) and health care cost data of veterans (aged ≥18 years) with positive test results for norovirus between January 1, 2010, and December 31, 2024. The University of Utah Institutional Review Board, the Centers for Disease Control and Prevention Human Research Protection Office, and the US Department of Veterans Affairs (VA) Salt Lake City Human Research Protection Program deemed this study exempt from review under exceptions 45 CFR 46.104(d)(4)(iv) and 45 CFR 46.101(b) and waived the informed consent requirement because the data used were obtained for the purpose of public health operations in the VHA; thus, the study met the requirements of public health surveillance as defined in 45CFR 46.102(I)(2). We followed the Consolidated Health Economic Evaluation Reporting Standards (CHEERS) reporting guideline.

EHR data were obtained from the VA Corporate Data Warehouse and the Praedico Public Health Surveillance System (Bitscopic Inc), which compiles public health data across multiple domains, including diagnostic testing.^13^ Health care cost data were obtained from the VA Managerial Cost Accounting System, an activity-based cost allocation system that maps expenditures from the VA payroll and general ledger to individual patient encounters.^14^ Costs were adjusted for inflation to 2024 dollars using the Personal Consumption Expenditures health price index.^15^

### Norovirus Episodes

Laboratory-confirmed norovirus episodes were defined as any norovirus-positive detection using polymerase chain reaction (PCR) assays conducted by internal VHA, commercial, or state public health laboratories. Multiple norovirus-positive PCR results per person were included; however, repeat norovirus-positive results within 30 days were assumed to be from the same episode and deduplicated by retaining the earliest test result. Due to manufacturer reports of potential false-positive results for norovirus in 2024,^16^ some results underwent confirmatory testing and were excluded if confirmed negative.

Norovirus episodes were exclusively categorized as outpatient, ED, or inpatient. Outpatient and ED episodes were defined as norovirus-positive PCR results from patients with no hospital admission within 7 days of the test date, with a clinic classification (stop code) used to differentiate ED from outpatient settings. Episodes that began in the outpatient or ED setting but required hospitalization were categorized as inpatient, and their outpatient and ED costs were included in their total costs. Community-acquired inpatient episodes were defined as norovirus-positive PCR results from patients with a hospital admission within 2 days before or after the test date. Hospital-acquired inpatient episodes were defined as norovirus-positive PCR results from patients admitted 3 days prior to the test and were excluded because norovirus-associated costs could not be separated from other costs prior to the norovirus episode. Norovirus-positive PCR results from patients admitted 3 to 7 days after the test date were also excluded because these admissions were less likely due to a current episode of norovirus.^17^

### Health Care Costs

For outpatient and ED episodes, costs for any encounter, laboratory, radiology or imaging, or pharmacy within 2 days of the norovirus-positive PCR results were included. A 2-day window was chosen to increase the likelihood that the health care costs were associated with the norovirus episode and not associated with other illnesses. For inpatient episodes, costs were obtained for the entire hospitalization and were stratified by laboratory, nursing, radiology or imaging, pharmacy, surgery, and other costs.

### Other Variables

Other variables extracted from the EHR included age; sex; race and ethnicity; rurality of home address^18^; *International Classification of Diseases, Ninth Revision, Clinical Modification* (*ICD-9-CM*) and *International Statistical Classification of Diseases, Tenth Revision, Clinical Modification* (*ICD-10-CM*) codes; and a patient’s problem list. Underlying medical conditions were based on *ICD-9-CM* and *ICD-10-CM* codes from hospitalizations that occurred prior to the date of norovirus-positive PCR results or from active conditions on a patient’s problem list. *ICD* codes were grouped into underlying condition categories based on the Charlson Comorbidity Index (CCI; score range: 0-29, with the highest score indicating that the patient had all medical conditions in the CCI) and into other relevant conditions based on Healthcare Cost and Utilization Project clinical classification.^19,20^ Race and ethnicity were primarily self-reported and categorized as Hispanic or Latino; non-Hispanic American Indian or Alaska Native, Asian, Black or African American, Native Hawaiian or Other Pacific Islander, and White; multiracial; and unknown or missing. Race and ethnicity data were collected to describe the demographics of the study population.

### Extrapolation to VHA Population and US Population 65 Years or Older 

Not all patients who seek care for acute gastroenteritis are tested for norovirus. We extrapolated median per-episode costs to the expected annual number of norovirus episodes in the VHA both with and without laboratory confirmation. The expected annual number of norovirus episodes with laboratory confirmation was calculated from the mean annual number of laboratory-confirmed norovirus episodes in the VHA between 2022 and 2024. The expected annual number of non–laboratory-confirmed norovirus episodes was calculated by subtracting the expected annual number of laboratory-confirmed episodes from the expected overall number of norovirus episodes in the VHA. This expected overall number was obtained by multiplying published norovirus incidence rates from the VA sentinel site surveillance by the VHA health care census data from 2024.^10,12,21^ The proportions of norovirus episodes with laboratory confirmation were approximately 5% in the outpatient, 12% in the ED, and 44% in the inpatient settings. For extrapolated costs, the median per-episode costs with and without laboratory costs were multiplied by the annual expected number of laboratory-confirmed and non–laboratory-confirmed norovirus episodes, respectively. We conducted sensitivity analyses to explore more conservative extrapolations by using the 25th percentile of costs, instead of median costs, and the lower 95% CI for norovirus incidence.

We also extrapolated total direct medical costs to the US population aged 65 years or older, using published incidence and annual count estimates.^4^ The proportions of norovirus episodes with laboratory confirmation for this US population were assumed to be similar to the proportions estimated for the VHA population using the same extrapolation methods.

### Statistical Analysis

Patient characteristics were summarized using numbers and proportions for categorical variables and medians with IQRs for continuous variables. Costs were summarized using medians (IQRs) and means (SDs). Median outpatient, ED, and inpatient costs were compared across age, race and ethnicity, and CCI using the Kruskal-Wallis test due to non-normality of the cost data, with a 2-sided *P* < .05 considered to be statistically significant. Positive test results, CCI, and costs were stratified by 3-year strata to assess temporal trends. Inpatient costs were assessed for patients with immunocompromised conditions, transplant, dementia, cerebrovascular disease, diabetes, kidney disease, chronic pulmonary disease, and peripheral vascular disease. SAS, version 9.4 (SAS Institute), was used for statistical analyses.

## Results

### Patient Characteristics

From 2010 through 2024, 8466 US veterans had 8743 laboratory-confirmed norovirus episodes and available data on health care costs. We excluded 908 hospital-acquired norovirus episodes and 67 episodes from patients who were admitted between 3 and 7 days after the norovirus-positive PCR result. Thus, 7768 norovirus episodes (among 7520 patients) were included in the analysis, consisting of 3520 in the outpatient, 2018 in the ED, and 2230 in the inpatient settings (Table 1).

**Table 1.** Characteristics of Patients With Medically Attended Norovirus Episodes

Characteristic	Norovirus episodes (n = 7768), No. (%)
All unique patients	7520
Age, y	
Median (IQR)	62 (45-74)
At time of test	
18-24	178 (2)
25-44	1744 (22)
45-64	2297 (30)
65-84	3068 (40)
≥85	481 (6)
Sex	
Female	906 (12)
Male	6862 (88)
Race and ethnicity^a^	
Hispanic or Latino	609 (8)
Non-Hispanic American Indian or Alaska Native	63 (1)
Non-Hispanic Asian	74 (1)
Non-Hispanic Black or African American	1041 (13)
Non-Hispanic Native Hawaiian or Other Pacific Islander	84 (1)
Non-Hispanic White	4953 (64)
Non-Hispanic multiracial	22 (0)
Unknown or missing	922 (12)
Rurality of patient home address	
Urban	5566 (72)
Rural	2132 (27)
Highly rural	64 (1)
Unknown	6 (0)
Setting of care	
Outpatient	3520 (45)
ED	2018 (26)
Inpatient	2230 (29)
Underlying medical conditions data available	7586 (98)
Charlson Comorbidity Index^b^	
0	3120 (41)
1-2	1878 (25)
3-4	1071 (14)
≥5	1517 (20)
Median (IQR)	1 (0-4)
Specific comorbidities	
Immunocompromised conditions^c^	1344 (18)
Transplant receipt	290 (4)
Dementia	436 (6)
Cerebrovascular disease	705 (9)
Diabetes	1989 (26)
Kidney disease	1484 (20)
Chronic pulmonary disease	1624 (21)
Peripheral vascular disease	816 (11)

Abbreviation: ED, emergency department.

^a^ Race and ethnicity were primarily self-reported and obtained from the Veterans Health Administration electronic health records.

^b^ Charlson Comorbidity Index is a summary of comorbid disease calculated as a weighted score that accounts for the quantity and severity of comorbidities; scores range from 0 to 29, with the highest score indicating that the patient had all medical conditions in the CCI. The following conditions were mutually exclusive: diabetes with chronic complications and diabetes without chronic complications, mild liver disease and moderate or severe liver disease, and any malignant neoplasm and metastatic solid tumor.

^c^ Immunocompromised conditions included HIV infection, malignant neoplasms and metastatic solid tumor history of transplant (*International Statistical Classification of Diseases, Tenth Revision [ICD-10]* codes D89.81, T86, Z94, Z98.85, Z48.2; *International Classification of Diseases, Ninth Revision [ICD-9]* code V42 996.8), immunosuppressive therapy (*International Statistical Classification of Diseases, Tenth Revision, Clinical Modification [ICD-10-CM]* codes Z92.25, Z79.899, Z79.6), and immunity disorders (*ICD-10* Healthcare Cost and Utilization Project Clinical Classification Software [CCS] = BLD008; *ICD-9* CCS = 3.10).

Of the 7768 norovirus episodes, 6862 (88%) were among male patients and 906 (12%) were among female patients with a median (IQR) age of 62 (45-74) years. Among 6846 norovirus episodes in patients with known race and ethnicity, 609 (8%) were among Hispanic or Latino, 63 (1%) among non-Hispanic American Indian or Alaska Native, 74 (1%) among non-Hispanic Asian, 1041 (13%) among non-Hispanic Black or African American, 84 (1%) among non-Hispanic Native Hawaiian or Other Pacific Islander, 4953 (64%) among non-Hispanic White, and 22 (0%) among multiracial veterans. A total of 1517 norovirus episodes (20%) were from patients with a CCI of 5 or greater, and the median (IQR) CCI was 1 (0-4).

### Outpatient Costs

Among 3520 outpatient norovirus episodes, the median (IQR) cost was $640 ($207-$1291) (Figure). Mean (SD) costs are presented in eTable 1 in Supplement 1. Outpatient costs differed by age, with the lowest median (IQR) cost among patients aged 85 years or older ($340 [$110-$965]) (Table 2). There were no statistically significant differences in outpatient costs by race and ethnicity, sex, rurality of home address, or CCI. Mean (SD) laboratory costs ($402 [$586]) accounted for 39% of the overall mean (SD) outpatient costs ($1037 [$1821]) (eTable 2 in Supplement 1).

**Figure.**
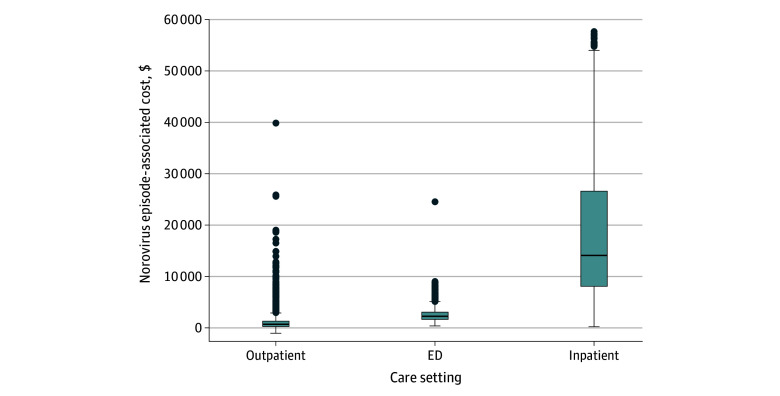
Costs Associated With Norovirus Episodes in the Inpatient, Emergency Department (ED), and Outpatient Settings at the Veterans Health Administration From 2010 to 2024 Costs adjusted to 2024 dollars. Upper and lower ends of the boxes represent the 25th and 75th percentiles (IQR), respectively; horizontal lines inside boxes represent the median; whiskers represent 1.5 times the IQR; and circles above boxes represent outliers beyond 1.5 times the IQR.

**Table 2.** Health Care Costs Associated With Outpatient, Emergency Department, and Inpatient Norovirus Episodes Stratified by Patient Characteristics

Characteristic	Outpatient			ED			Inpatient		
	No.	Median cost (IQR), US $^a^	*P* value^b^	No.	Median cost (IQR), US $^a^	*P* value^b^	No.	Median cost (IQR), US $^a^	*P* value^b^
Overall	3520	640 (207-1291)	NA	2018	2203 (1596-2989)	NA	2230	14 083 (8045-26 672)	NA
Age, y									
18-24	120	401 (199-665)	<.001	39	2394 (1574-2984)	.56	19	7751 (4768-14 984)	<.001
25-44	796	721 (263-1369)		661	2171 (1609-2927)		287	9394 (5474-15 830)	
45-64	1017	753 (290-1343)		681	2231 (1598-3010)		599	12 777 (7297-23 737)	
65-84	1400	590 (173-1269)		594	2235 (1592-3047)		1074	16 075 (9291-29 311)	
≥85	187	340 (110-965)		43	1873 (1468-2665)		251	18 566 (10 676-33 582)	
Race and ethnicity^c^									
Hispanic or Latino	250	731 (240-1418)	.22	205	2396 (1751-3089)	.02	154	12 880 (6628-19 970)	.06
Non-Hispanic Black or African American	465	732 (228-1267)		265	2125 (1562-2796)		311	14 144 (7810-26 798)	
Non-Hispanic White	2251	618 (195-1310)		1221	2155 (1574-2989)		1481	14 609 (8413-26 996)	
Other or unknown^d^	554	592 (206-1241)		327	2237 (1621-3042)		284	13 279 (7356-26 858)	
Sex									
Female	492	672 (162-1274)	.36	256	2176 (1564-2965)	.82	158	12 558 (6280-23 500)	.01
Male	3028	636 (216-1296)		1762	2204 (1598-2991)		2072	14 196 (8234-26 772)	
Rurality of patient home address									
Urban	2448	650 (206-1293)	.52	1499	2204 (1576-2981)	.98	1619	13 718 (7810-25 682)	.07
Rural	1043	616 (214-1276)		507	2206 (1617-3005)		582	15 147 (8521-28 807)	
Highly rural	27	524 (173-1230)		9	1893 (1744-3046)		28	14166 (9765-23 038)	
Unknown	2	1690 (899-2481)		3	2125 (1402-3095)		0	NA	
Charlson Comorbidity Index									
0	1644	685 (239-1275)	.12	1112	2172 (1576-2936)	.004	364	9401 (5714-15 836)	<.001
1-2	825	648 (188-1290)		488	2142 (1596-2888)		565	12 164 (7213-21 010)	
3-4	417	523 (181-1184)		181	2242 (1546-3113)		473	14 921 (8695-27 559)	
≥5	513	626 (197-1539)		178	2542 (1681-3488)		826	18 508 (10 445-35 900)	
Unknown	121	412 (142-747)		59	2432 (1631-3043)		2	16 619 (6015-27 223)	

Abbreviations: ED, emergency department; NA, not applicable.

^a^ Costs adjusted to 2024 dollars.

^b^ Calculated using the Kruskal-Wallis test.

^c^ Race and ethnicity were primarily self-reported and obtained from the Veterans Health Administration electronic health records.

^d^ Other included non-Hispanic American Indian or Alaska Native, non-Hispanic Asian, and multiracial.

### ED Costs

Among 2018 ED norovirus episodes, the median (IQR) cost was $2203 ($1596-$2989) (Figure). There were no statistically significant differences in ED costs by age, sex, or rurality of home address. ED costs differed by race and ethnicity, with the highest median (IQR) cost among Hispanic or Latino patients ($2396 [$1751-$3089]) (Table 2). ED costs also differed by CCI, with the highest median (IQR) cost among patients with a CCI of 5 or higher ($2542 [$1681-$3488]) (Table 2). Mean (SD) laboratory costs ($527 [$484]) accounted for 22% of the overall mean (SD) ED costs ($2436 [$1305]) (eTable 2 in Supplement 1).

### Inpatient Costs

Among 2230 inpatient norovirus episodes, the median (IQR) cost was $14 083 ($8045-$26 672) (Figure). The median (IQR) length of stay was 3 (2-5) days. The median (IQR) cost per day was $4903 ($3731-$6508). Inpatient costs expectedly increased with longer hospital stays, with a median (IQR) cost of $58 378 ($40 018-$94 663) for patients staying 7 or more days (eTable 3 in Supplement 1). Median (IQR) inpatient costs were higher among older age groups ($12 777 [$7297-$23 737], $16 075 [$9291-$29 311], and $18 566 [$10 676-$33 582] for those aged 45-64 years, 65-84 years, and ≥85 years, respectively) than younger adults ($7751 [$4768-$14 984] and $9394 [$5474-$15 830] for those aged 18-24 years and 25-44 years, respectively; *P* < .001) (Table 2). The length of stay was shorter among younger than older adults, but when restricted to patients with a consistent length of stay (eg, 3 days), those aged 18 to 24 years and 25 to 44 years still had slightly lower median (IQR) costs than those 45 years or older (eg, $6587 [$4643-$7751] and $7732 [$5043-12 048] vs $9620 [$6445-$13 742]) (eTable 4 in Supplement 1). There were no statistically significant differences in inpatient costs by race and ethnicity or rurality of home address.

Inpatient costs increased with higher CCI, with the highest median (IQR) cost among patients with a CCI of 5 or higher ($18 508 [$10 445-$35 900]). Patients who received transplants had higher median (IQR) costs ($18 715 [$9703-$36 382]; *P* = .002) than those without transplants ($13 797 [$8030-$26 044]). Median (IQR) costs were high among patients with dementia ($20 776 [$11 385-$38 111]), cerebrovascular disease ($17 001 [$9751-$33 582]), diabetes ($15 383 [$8559-$28 826]), kidney disease ($16 410 [$9832-$33 444]), chronic pulmonary disease ($16 477 [$10 100-$29 884]), and peripheral vascular disease ($17 520 [$11 137-$34 990]) (eTable 3 in Supplement 1). Forty-eight percent of overall inpatient costs were categorized as mean (SD) bed day or nursing costs ($11 764 [$21 203]), and 4% of overall inpatient costs were attributed to mean (SD) laboratory costs ($1064 [$1829]) (eTable 2 in Supplement 1).

### Trends Over Time

Due to increased availability of norovirus testing in the VHA, norovirus-positive episodes increased over time (Table 3). In the outpatient setting, the median (IQR) CCI of patients with norovirus-positive episodes decreased over time, from 3 (1-5) in the 2010-2012 period to 1 (0-2) in the 2022-2024 period. There tended to be slightly increased median (IQR) overall costs and laboratory costs over time in all settings.

**Table 3.** Temporal Trends of Norovirus-Positive Episodes, Charlson Comorbidity Index, and Costs

Study period	Norovirus-positive episodes, No. (%)	Median (IQR)		
		CCI	Overall costs, $^a^	Laboratory costs, $^a^
**Outpatient**				
2010-2012	119 (3)	3 (1-5)	248 (117-425)	52 (43-156)
2013-2015	105 (3)	3 (1-5)	342 (149-543)	168 (76-351)
2016-2018	404 (11)	2 (0-5)	551 (172-1081)	193 (99-444)
2019-2021	544 (16)	0 (0-1)	675 (259-1433)	265 (91-911)
2022-2024	2348 (67)	1 (0-2)	729 (228-1334)	287 (86-767)
**ED**				
2010-2012	24 (1)	0 (0-2)	1215 (983-1345)	180 (118-265)
2013-2015	30 (1)	1 (0-2)	1448 (1099-1782)	279 (201-397)
2016-2018	233 (2)	1 (0-2)	1624 (1105-2176)	304 (193-633)
2019-2021	411 (20)	0 (0-1)	2298 (1709-3043)	396 (223-904)
2022-2024	1320 (65)	0 (0-2)	2325 (1708-3088)	324 (187-810)
**Inpatient**				
2010-2012	114 (5)	3 (1-5)	11 429 (6523-22 527)	541 (335-963)
2013-2015	140 (6)	3 (1-5)	14 350 (8293-25 736)	562 (337-871)
2016-2018	264 (12)	3 (1-6)	12 363 (7165-21 830)	556 (308-1019)
2019-2021	301 (14)	3 (1-5)	15 032 (9187-28 130)	736 (326-1803)
2022-2024	1411 (63)	3 (1-6)	14 487 (8199-28 097)	583 (256-1257)

Abbreviations: CCI, Charlson Comorbidity Index; ED, emergency department.

^a^ Costs adjusted to 2024 dollars.

### Extrapolated Costs

The extrapolated cost using published norovirus incidence rates, stratified by expected laboratory-confirmed and non–laboratory-confirmed episodes, was $28 438 556 annually (Table 4). Sensitivity analyses using more conservative per-episode costs and lower norovirus incidence rates resulted in $14 322 626 to $21 241 946 in extrapolated costs to the VHA population (eTable 5 in Supplement 1). The extrapolated cost to the US population 65 years or older was $847 776 600, with more conservative estimates of $444 617 160 to $610 439 880 (eTable 5 in Supplement 1).

**Table 4.** Extrapolated Annual Costs of Norovirus Episodes in the Veterans Health Administration Population and US Population 65 Years or Older

	Per-episode cost, median (IQR), $^a^	VHA population		US population aged ≥65 y	
		Expected annual number^b^	Extrapolated cost, US $^c^	Expected annual number^d^	Extrapolated cost, US $^c^
**Outpatient**					
Laboratory-confirmed	640 (207-1291)	825	528 213	21 200	13 568 000
Non–laboratory-confirmed	220 (0-707)	15 788	3 473 328	402 800	88 616 000
Overall outpatient	NA	16 613	4 001 541	424 000	102 184 000
**ED**					
Laboratory-confirmed	2203 (1596-2989)	440	969 320	7200	15 861 600
Non–laboratory-confirmed	1666 (1209-2303)	3084	5 137 961	52 800	87 964 800
Overall ED	NA	3524	6 107 281	60 000	103 826 400
**Inpatient**					
Laboratory-confirmed	14 083 (8045-26 672)	589	8 294 887	20 680	291 236 440
Non–laboratory-confirmed	13 318 (7543-25 340)	753	10 034 847	26 320	350 529 760
Overall inpatient	NA	1342	18 329 734	47 000	641 766 200
Total	NA	NA	28 438 556	NA	847 776 600

Abbreviations: ED, emergency department; NA, not applicable; VHA, Veterans Health Administration.

^a^ The median cost per episode (adjusted to 2024 dollars) for non–laboratory-confirmed cases was equal to the median costs excluding laboratory costs for the VHA population.

^b^ Expected annual counts for the VHA population were obtained from multiplying incidence rates by the 2024 VHA health care census data.^21^ Incidence rates per 100 000 people were obtained from Cardemil et al.^10^ Outpatient and ED incidence rates were combined in this publication; thus, the approximately 5:1 ratio of outpatient to ED episodes in Burke et al^4^ was applied to these incidence rates to obtain stratified outpatient and ED rates. The expected annual number of norovirus episodes with laboratory confirmation was calculated from the mean annual number of laboratory-confirmed norovirus episodes in the VHA between 2022 and 2024. Norovirus episodes with laboratory confirmation were approximately 5% in the outpatient setting, 12% in the ED setting, and 44% in the inpatient setting.

^c^ Extrapolated costs were equal to the expected annual numbers multiplied by the median per-episode cost, stratified by laboratory confirmation and excluding laboratory costs, for those without laboratory confirmation.

^d^ Expected annual numbers for the US population aged 65 years or older were obtained from Burke et al.^4^ Norovirus episodes with laboratory confirmation in the VHA population were applied to the US population: approximately 5% in the outpatient setting, 12% in the ED setting, and 44% in the inpatient setting.

## Discussion

Using a large, national database of adult veterans, we estimated median costs of $14 083 for inpatient, $2203 for ED, and $640 (in 2024 dollars) for outpatient laboratory-confirmed norovirus episodes. The overall extrapolated annual costs were approximately $28 million in the VHA population and approximately $848 million in the US population aged 65 years or older. These costs illustrate a substantial economic burden and, to our knowledge, provide the first measures of health care costs of laboratory-confirmed norovirus episodes in an adult US population.

Health care costs were highest for norovirus episodes that required hospitalization. Our inpatient cost estimate of $14 083 is similar to norovirus hospitalization costs of approximately $10 000 (in 2020 dollars) and $8000 to $9000 (in 2007 dollars) from modeling studies.^5,6^ These costs are also within the range of cost estimates for *Clostridioides difficile* infection, a bacterial cause of severe diarrhea with a high incidence in this hospitalized population.^10,22,23^ We found a pattern of increasing inpatient costs with increasing age and increasing CCI. Age-related changes in B-cell and T-cell function and immunosenescence, as well as complications from underlying conditions, may necessitate longer hospitalizations or more complex and costly health care needs. Patients who received a transplant are especially vulnerable to norovirus and longer hospitalizations given their immunocompromised condition.^24^ We found that transplant recipients with norovirus episodes had higher inpatient costs than patients without a transplant. There are currently no antivirals against norovirus; thus, treatment for both patients with and without immunocompromised conditions relies on clinical management of symptoms and rehydration.^25^ Our findings illustrate the potential cost savings if future antivirals could reduce symptom length or severity and subsequently length of hospital stay.

While the median costs for outpatient and ED norovirus episodes were approximately 22 and 6 times less than the median inpatient costs, the extrapolated annual costs were substantial due to the higher incidence of medically attended norovirus in these settings. Unexpectedly, the median outpatient and ED costs were lowest among patients 85 years or older; the reason for this finding is unclear, but it is possible that more of these individuals reside in nursing homes or skilled nursing facilities, which may incur a greater burden of their medical management than the VHA medical facilities. ED costs increased with increasing CCI, but outpatient costs were consistent across CCI categories, indicating minimal implications of underlying conditions for the costs accrued in an outpatient setting.

The extrapolated costs associated with outpatient, ED, and inpatient norovirus episodes among adults illustrate a substantial annual economic burden on the VHA and the entire US health care system. Currently, the only available interventions to prevent norovirus episodes are nonpharmaceutical, including appropriate hand-hygiene and infection-control prevention measures. Decades of research and preliminary clinical trials have led to progress in the development of norovirus vaccines and antivirals; however, the road to an approved effective vaccine or antiviral has been challenging.^8,26,27,28^ Adults 65 years or older may be a future target population for vaccines and therapeutics due to the clinical outcomes in this older population and the economic burden as evidenced by this study.

### Limitations

There are certain limitations that should be considered in the interpretation of this data. Patients who undergo laboratory testing may have more severe illness and associated costs than those who do not, especially in the outpatient setting. However, laboratory testing for norovirus has become more available in recent years, including in the VHA. In this study, there was an increase over time in norovirus-positive episodes in the outpatient setting among healthier individuals with fewer comorbidities, suggesting that testing may now be more common for less severe cases and thus increasing the generalizability of the study population and findings. Because there may still be an overrepresentation of more severe illness among those who are tested, we considered conservative assumptions where possible in our extrapolations. Even these more conservative assumptions resulted in substantial economic burden. Additionally, these extrapolated costs do not account for societal costs or facility-level costs accrued during outbreaks at nursing homes or other congregate settings, likely underestimating overall norovirus-associated costs.^5^ Patients in the VHA also may not be representative of the entire US adult population, as veterans are generally older and more likely to be male compared with other patients in US hospitals.^11^ The financing and accounting for VHA health care facilities differ from those for non-VHA facilities. However, there is some, albeit limited, evidence that costs of providing health care may be lower in the VHA than non-VHA facilities, suggesting our extrapolations were conservative.^29,30^ Our findings of $28 million direct VHA annual medical costs provide valuable economic cost data for the VHA that were otherwise missing from prior cost analyses. The database structure of the VA Managerial Cost Accounting System did not allow for isolation of costs associated with hospital-acquired norovirus episodes, which may have different inpatient costs from community-acquired norovirus episodes, and represented 10% of all laboratory-confirmed cases in this study. While health care costs were assumed to be associated with norovirus if they were incurred within 2 days of a norovirus-positive test result, we cannot confirm that norovirus directly produced these health care costs. However, this short-time interval was chosen to increase the probability that the encounters and associated costs were related to norovirus.

## Conclusions

This study provided a comprehensive summary of outpatient, ED, and inpatient costs associated with laboratory-confirmed norovirus episodes among US veterans seeking health care in the VHA. While norovirus is generally considered a mild acute illness, the findings highlight its substantial economic burden and potential cost savings from targeted interventions, such as vaccines and antivirals, for this adult population.

## Notes

Supplement 1. **eTable 1.** Mean (SD) Costs Associated With Outpatient, Emergency Department (ED), and Inpatient Norovirus Episodes Stratified by Patient Characteristics, Veterans Health Administration 2010-2024 (in 2024 U.S. Dollars) **eTable 2.** Costs for Norovirus-Episodes Stratified by Cost Type, Veterans Health Administration, 2010-2024 (in 2024 US Dollars) **eTable 3.** Health Care Costs Associated With Community-Acquired Inpatient Norovirus Episodes Stratified by Additional Patient Characteristics, Veterans Health Administration, 2010-2024 (in 2024 U.S. Dollars) **eTable 4.** A) Length of Inpatient Stay by Age Group and B) Inpatient Costs (in 2024 U.S. Dollars) by Age Group When Restricted to Patients With a 3-Day (the Median) Length of Stay, Veterans Health Administration, 2010-2024 **eTable 5.** Sensitivity Analysis Results for Extrapolation to Veterans Health Administration (VHA) Population, and US Population Aged 65-Years and Older
eReferences

Supplement 2.
Data Sharing Statement
