# Supplementary material for: Health Care Costs Associated With Norovirus at the Veterans Health Administration
Source: JAMA Netw Open. 2025 Oct 9;8(10):e2536600. doi: 10.1001/jamanetworkopen.2025.36600 (PMC12511989; doi:10.1001/jamanetworkopen.2025.36600)
Supplement: Supplement 1. — eTable 1. Mean (SD) Costs Associated With Outpatient, Emergency Department (ED), and Inpatient Norovirus Episodes Stratified by Patient Characteristics, Veterans Health Administration 2010-2024 (in 2024 U.S. Dollars) eTable 2. Costs for Norovirus-Episodes Stratified by Cost Type, Veterans Health Administration, 2010-2024 (in 2024 US Dollars) eTable 3. Health Care Costs Associated With Community-Acquired Inpatient Norovirus Episodes Stratified by Additional Patient Characteristics, Veterans Health Administration, 2010-2024 (in 2024 U.S. Dollars) eTable 4. A) Length of Inpatient Stay by Age Group and B) Inpatient Costs (in 2024 U.S. Dollars) by Age Group When Restricted to Patients With a 3-Day (the Median) Length of Stay, Veterans Health Administration, 2010-2024 eTable 5. Sensitivity Analysis Results for Extrapolation to Veterans Health Administration (VHA) Population, and US Population Aged 65-Years and Older eReferences [file jamanetwopen-e2536600-s001.pdf]

Supplemental Online Content

Cates JE, Nelson RE, Suo Y, et al. Health care costs associated with norovirus at the Veterans Health Administration. *JAMA Netw Open*. 2025;8(10):e2536600. doi:10.1001/jamanetworkopen.2025.36600

eTable 1. Mean (SD) Costs Associated With Outpatient, Emergency Department (ED), and Inpatient Norovirus Episodes Stratified by Patient Characteristics, Veterans Health Administration 2010−2024 (in 2024 U.S. Dollars)

eTable 2. Costs for Norovirus-Episodes Stratified by Cost Type, Veterans Health Administration, 2010−2024 (in 2024 U.S. Dollars)

eTable 3. Health Care Costs Associated With Community-Acquired Inpatient Norovirus Episodes Stratified by Additional Patient Characteristics, Veterans Health Administration, 2010−2024 (in 2024 U.S. Dollars)

eTable 4. A) Length of Inpatient Stay by Age Group and B) Inpatient Costs (in 2024 U.S. Dollars) by Age Group When Restricted to Patients With a 3-Day (the Median) Length of Stay, Veterans Health Administration, 2010−2024

eTable 5. Sensitivity Analysis Results for Extrapolation to Veterans Health Administration (VHA) Population, and U.S. Population Aged 65-Years and Older

eReferences

This supplemental material has been provided by the authors to give readers additional information about their work.

© 2025 Cates JE et al. *JAMA Network Open*

eTable 1. Mean (SD) costs associated with outpatient, emergency department (ED), and inpatient norovirus episodes stratified by patient characteristics, Veterans Health Administration 2010−2024 (in 2024 U.S. dollars).

	Outpatient		ED		Inpatient	
	**N**	**Mean (SD), $**	**N**	**Mean (SD), $**	**N**	**Mean (SD), $**
**Overall**	3520	1037 (1821)	2018	2436 (1305)	2230	24 670 (37321)
**Age**, years						
18–24	120	567 (579)	39	2371 (986)	19	11 576 (9888)
25–44	796	1062 (1370)	661	2397 (1169)	287	14 626 (19 489)
45–64	1017	1076 (1600)	681	2465 (1469)	599	22 333 (32 175)
65–84	1400	1070 (2279)	594	2461 (1256)	1074	28 196 (44 400)
≥ 85	187	762 (1121)	43	2299 (1478)	251	27 635 (28 915)
**Race, ethnicity**						
Black, non-Hispanic 4	465	1112 (1642)	265	2305 (1089)	311	26 201 (39 086)
Hispanic or Latino 5	250	1194 (2926)	205	2584 (1177)	154	22 374 (37 869)
White, non-Hispanic 6	2251	1046 (1849)	1221	2418 (1371)	1481	24 614 (37 603)
Other[a] or unknown 1	554	862 (1022)	327	2519 (1282)	284	24 533 (33 497)
**Sex**						
Female	492	936 (1363)	256	2504 (1843)	158	18 770 (24 078)
Male	3028	1053 (1885)	1762	2427 (1207)	2072	25 120 (38 110)
**Rurality of patient home address**						
Urban	2448	1021 (1821)	1499	2451 (1360)	1619	24 201 (36 250)
Rural	1043	1070 (1815)	507	2394 (1133)	582	26 152 (40 440)
Highly Rural	27	1123 (2169)	9	2558 (1133)	28	21 490 (31 235)
Unknown	2	1690 (1119)	3	2207 (849)	0	NA
**Charlson Comorbidity Index**						
0	1644	1002 (1448)	1112	2371 (1153)	364	13 427 (14 631)
1–2	825	933 (1092)	488	2431 (1555)	565	18 562 (20 785)
3−4	417	1204 (2835)	181	2449 (1228)	473	25 267 (36 951)
≥5	513	1272 (2712)	178	2803 (1463)	826	33 480 (49 325)
Unknown	121	641 (764)	59	2563 (1315)	2	16 619 (14 996)

SD=Standard deviation, NA=not applicable

[a] Other included non-Hispanic American Indian or Alaska Native, non-Hispanic Asian, and multiracial

© 2025 Cates JE et al. *JAMA Network Open*

eTable 2. Costs for norovirus-episodes stratified by cost type, Veterans Health Administration, 2010−2024 (in 2024 U.S. dollars).

		Costs, $		Costs among those with any costs in that category, $		Average proportion of overall costs
	**No.**	**Mean (SD)**	**Median (IQR)**	**N**	**Median (IQR)**	**%**
**Outpatient**						
**Overall**	3520	1037 (1821)	640 (207−1291)	3520	640 (207−1291)	100
Laboratory	3520	402 (586)	178 (34−644)	3031	257 (87−739)	39
**ED**						
**Overall**	2018	2436 (1305)	2203 (1596−2989)	2018	2203 (1596−2989)	100
Laboratory	2018	527 (484)	327 (189−785)	1997	334 (193−788)	22
**Inpatient**						
**Overall**	2230	24 670 (37 321)	14 083 (8045−26 672)	2230	14 083 (8045−26 672)	100
**Cost Category** [a]						
Laboratory	2230	1064 (1829)	574 (253−1217)	2158	595 (280−1254)	4
Bed day/Nursing	2230	11 764 (21 203)	6205 (3399−11 902)	2160	6457 (3683−12 217)	48
Other	2230	8301 (12 544)	4487 (2293−9220)	2227	4498 (2303−9225)	34
Radiology	2230	601 (1797)	0 (0−546)	1034	611 (310−1326)	2
Pharmacy	2230	2272 (3893)	1131 (523−2396)	2166	1198 (567−2472)	9
Surgery	2230	504 (3228)	0 (0−0)	356	729 (362−1682)	2
Outpatient or ED costs prior to hospitalization	2230	165 (879)	0 (0-0)	106	3181 (1892−4596)	1

[a] Laboratory includes all laboratory departments; Bed day/nursing includes wards-nursing, observation wards, and psychiatry residential rehab; radiology includes general radiology, nuclear medicine, and radiation therapy; pharmacy includes all pharmacy departments; surgery includes surgery and anesthesiology; all other costs are assigned to the 'other' category.[3]

© 2025 Cates JE et al. *JAMA Network Open*

eTable 3. Health care costs associated with community-acquired inpatient norovirus episodes stratified by additional patient characteristics, Veterans Health Administration, 2010−2024 (in 2024 U.S. dollars).

	N	Median (IQR), $	*P* value[a]
**Overall**	2230	14 083 (8045–26 672)	
**Specific medical conditions**			
Immunocompromising condition [b]	657	16 599 (9176–31 288)	<.001
Transplant recipient	122	18 715 (9703–36 382)	0.002
Dementia	226	20 776 (11 385–38 111)	<.001
Cerebrovascular disease	327	17 001 (9751–33 582)	<.001
Diabetes	863	15 383 (8559–28 826)	0.0003
Renal disease	764	16 410 (9832–33 444)	<.001
Chronic pulmonary disease	714	16 477 (10 100–29 884)	<.001
Peripheral vascular disease	387	17 520 (11 137–34 990)	<.001
**Length of stay strata**			
1–3 days	1346	9251 (6066–13 359)	<.001
4–7	553	23 669 (18 004–30 518)	
>7	331	58 378 (40 018-94 663)	

[a] Kruskal–Wallis test

[b] HIV, any malignancy, metastatic malignancy, immunity disorder, immunosuppressive therapy

© 2025 Cates JE et al. *JAMA Network Open*

eTable 4. A) Length of inpatient stay by age group and B) inpatient costs (in 2024 U.S. dollars) by age group when restricted to patients with a 3-day (the median) length of stay, Veterans Health Administration, 2010−2024.

	A) Length of stay					B) Inpatient costs, restricted to 3-day length of stay		
	Median (IQR), days	No. (%[a])	No. (%)	No. (%)				
		1−3 days	4−7 days	>7 days	*P* value[b]	N	Median (IQR), $	*P* value[b]
**Age**, years								
18–24	3 (2–4)	13 (68)	4 (21)	2 (11)	<.001	13	6587 (4643–7751)	<.001
25–44	2 (1–3)	233 (81)	36 (13)	18 (6)		233	7732 (5043–12 048)	
45–64	2 (1–5)	392 (65)	131 (22)	76 (13)		392	8539 (6027–12 843)	
65–84	3 (2–6)	596 (55)	300 (28)	178 (17)		596	9949 (6640–14 636)	
≥ 85	4 (2–7)	112 (45)	82 (33)	57 (23)		112	10 372 (7209–13 150)	

IQR=interquartile range
[a] Row percent
[b] Kruskal–Wallis test

© 2025 Cates JE et al. *JAMA Network Open*

eTable 5. Sensitivity analysis results for extrapolation to Veterans Health Administration (VHA) population, and U.S. population aged 65-years and older. Sensitivity analysis 1 assumed a lower incidence of norovirus obtained from the lower 95% confidence interval of incidences reported in the respective references[1,2]. Sensitivity analysis 2 assumed lower per-episode costs using the 25th percentile of per-episodes costs rather than the 50th percentile (median).

	**Extrapolated costs**		
	**Main approach**	**Sensitivity Analysis 1: lower incidence**	**Sensitivity Analysis 2: lower per-episode cost**
**VHA**			
Outpatient	$4 001 541	$3 185 155	$170 844
ED	$6 107 281	$4 795 888	$3 729 777
Inpatient	$18 329 734	$13 260 903	$10 422 005
**Total**	**$28 438 556**	**$21 241 946**	**$14 322 626**
**U.S. over-65**			
Outpatient	$102 184 000	$73 505 000	$4 388 400
ED	$103 826 400	$72 678 480	$75 326 400
Inpatient	$641 766 200	$464 256 400	$364 902 360
Total	**$847 776 600**	**$610 439 880**	**$444 617 160**

ED=emergency department

© 2025 Cates JE et al. *JAMA Network Open*

eReferences

1. Cardemil CV, Balachandran N, Kambhampati A, et al. Incidence, etiology, and severity of acute gastroenteritis among prospectively enrolled patients in 4 veterans affairs hospitals and outpatient centers, 2016-2018. *Clin Infect Dis*. Nov 2 2021;73(9):e2729-e2738. doi:10.1093/cid/ciaa806
2. Burke RM, Mattison CP, Pindyck T, et al. Burden of norovirus in the united states, as estimated based on administrative data: Updates for medically attended illness and mortality, 2001-2015. *Clin Infect Dis*. Jul 1 2021;73(1):e1-e8. doi:10.1093/cid/ciaa438
3. Phibbs CS BP, Fan A. Research guide to the managerial cost accounting national cost extracts. *Menlo Park, CA VA Palo Alto, Health Economics Resource Center*. 2015;
