# Supplementary material for: Health Care Costs Associated With Norovirus at the Veterans Health Administration
Source: JAMA Netw Open. 2025 Oct 9;8(10):e2536600. doi: 10.1001/jamanetworkopen.2025.36600 (PMC12511989; doi:10.1001/jamanetworkopen.2025.36600)
Supplement: Supplement 2. — Data Sharing Statement [file jamanetwopen-e2536600-s002.pdf]

Data Sharing Statement

Cates. Health Care Costs Associated With Norovirus at the Veterans Health Administration.
JAMA Netw Open. Published October 09, 2025. doi:10.1001/jamanetworkopen.2025.36600

Data
Data available: No
